# Valganciclovir as Add-on to Second-Line Therapy in Patients with Recurrent Glioblastoma

**DOI:** 10.3390/cancers14081958

**Published:** 2022-04-13

**Authors:** Mattia Russel Pantalone, Afsar Rahbar, Cecilia Söderberg-Naucler, Giuseppe Stragliotto

**Affiliations:** 1 Microbial Pathogenesis Unit, Department of Medicine, Karolinska Institutet, 17164 Solna, Sweden; afsar.rahbar@ki.se (A.R.); giuseppe.stragliotto@regionstockholm.se (G.S.); 2 Department of Neurology, Karolinska University Hospital, 17177 Stockholm, Sweden; 3 MediCity Research Laboratory, University of Turku, 20520 Turku, Finland; 4 Institute of Biomedicine, University of Turku, 20520 Turku, Finland

**Keywords:** neuro-oncology, glioblastoma, cytomegalovirus, neurosurgery, valganciclovir

## Abstract

**Simple Summary:**

Patients with glioblastoma have a dismal prognosis. The major challenge with this disease is that it recurs despite aggressive first-line therapy and rapidly becomes therapy resistant. Cytomegalovirus has been found in most glioblastoma tumors and may contribute to tumor aggressiveness. Antiviral therapy may thus represent a novel therapeutic strategy and has shown promising results in patients with newly diagnosed glioblastoma. We performed a retrospective analysis of survival data of 29 patients with recurrent glioblastoma receiving the antiviral drug valganciclovir as an add-on to second-line therapy and of 109 contemporary controls treated at our institution. Valganciclovir was well tolerated and seemed to improve survival after tumor recurrence in patients with recurrent disease both in re-operated and non-re-operated patients and in patients with unmethylated and methylated MGMT promoter status. Prospective controlled clinical studies on patients with recurrent glioblastoma are warranted to evaluate if valganciclovir treatment offers a novel therapeutic option.

**Abstract:**

Glioblastoma invariably recurs despite aggressive and multimodal first-line treatment and no standardized second-line therapy exists. We previously reported that treatment with the antiviral drug valganciclovir as an add-on to standard therapy significantly prolonged overall survival in 102 patients with newly diagnosed glioblastoma compared to contemporary controls. Here we present the results of retrospective survival analyses including patients with glioblastoma that initiated valganciclovir therapy after recurrence. Twenty-nine patients with recurrent glioblastoma received valganciclovir as an add-on to second-line therapy at Karolinska University Hospital. Contemporary controls were 109 patients with glioblastoma who received similar second-line therapy at our institution. We retrospectively analyzed survival data of these patients. Patients with recurrent glioblastoma who received valganciclovir had longer median overall survival after recurrence than controls (12.1 vs. 7.4 months, respectively, *p* = 0.0028). The drug was well tolerated. Both patients who underwent re-operation and patients that were not re-operated after recurrence benefitted significantly from valganciclovir therapy. Valganciclovir prolonged survival after recurrence both in patients with an unmethylated and methylated MGMT promoter gene. Valganciclovir was safe to use and prolonged median survival after recurrence for patients with recurrent glioblastoma, re-operated or not after recurrence, and with methylated or unmethylated MGMT promoter gene.

## 1. Introduction

Glioblastoma (GBM) is the deadliest and most common primary brain tumor in adults with a median overall survival (OS) of only approximately 13–15 months [1,2]. Despite aggressive treatment, such as surgical resection followed by radiotherapy and temozolomide therapy, anti-angiogenic therapy (bevacizumab), or immunotherapy, the limited success of current treatment protocols leads to very poor prognosis. GBM tumors generally become therapy resistant and recur almost invariably after about 7 months [2]. Patients with GBM recurrence only survive about 3 months without therapy and 6–9 months with maximal second-line therapy [3,4]. As therapy success is lacking among patients with recurrent GBM, there is no consensus on treatment of recurrent GBM. Although it is undoubtedly effective in newly diagnosed GBM, surgical re-operation of recurrent GBM generally fails to improve survival in recurrent cases [1,5]. However, patients with recurrence should always be evaluated individually for re-operation since it may be effective in selected cases [6,7]. Second-line chemotherapy often includes a nitrosurea-based therapy (lomustine) that confers a progression-free survival rate at 6 months of 19%, but the objective response rate is less than 10% [8]. The anti-angiogenetic drug, bevacizumab, has not improved overall survival in either newly diagnosed or in recurrent GBM [9,10,11,12,13]. A temozolomide re-challenge has not led to consistent results; therefore, it is unclear whether it is beneficial for patients with recurrent GBM [14,15,16]. Re-irradiation may result in local disease control in a subgroup of patients [17,18,19,20,21], but this approach is burdened by high risk of neurotoxicity. Thus, due to the consistent failures in obtaining high benefits from individual therapy protocols for patients with recurrent GBM, there is no standardized effective treatment option for these patients.

The end-of-life phase of patients with GBM occurs when the tumor has become resistant to the available therapies and is characterized by an inexorable decline of cognitive and motor functions with palliative care as the only resource to offer some help to these patients [22].

In 2002, Cobbs reported that 100% of GBMs were infected with cytomegalovirus (CMV) [23]. This virus may affect the oncogenic potential of cells and can cause all the hallmarks of cancer [24]. We found that patients with low-grade CMV infection had longer overall survival (OS) than those with high-grade infection (33 vs. 13 months, *p* = 0.036) and they had a higher 2-year survival rate (63.6% vs. 17.2%, *p* = 0.003) [25,26]. These observations imply that CMV may affect tumor progression and that the virus could be a new target of therapy for patients with GBM.

To determine whether antiviral therapy against CMV was safe to use and potentially could improve the outcome of patients with GBM, we enrolled 42 patients in a double-blind clinical phase I/II pilot trial to assess the safety and potential efficacy of valganciclovir treatment as an add-on therapy in patients with newly diagnosed GBM (VIGAS, initiated in 2006) [27]. The study was underpowered and failed its primary end point of reduced tumor growth by MRI at 6 months. However, in exploratory analyses, we observed that VIGAS patients who were treated with valganciclovir had a better prognosis; 50% of valganciclovir-treated patients were alive at 2 years compared to 18% of contemporary control patients. The median OS was also longer in valganciclovir-treated patients than in controls (24.1 vs. 13.7 months, *p* = 0.0031) [27]. In a follow up analysis of 50 patients who were treated with valganciclovir as an add-on to therapy, we further observed a similar highly-improved survival rate [28]. As targeting CMV with valganciclovir supported prolonged survival of patients with newly diagnosed GBM, CMV-targeted therapy should be evaluated in controlled studies, to determine if it has a place in GBM treatment.

After completing the VIGAS trial and while awaiting funding to be granted to perform a clinical randomized controlled study to evaluate this therapy in patients with GBM, we offered valganciclovir to patients with GBM who requested this therapy as a compassionate therapy. The patients received valganciclovir in addition to their standard treatment, and most were treated at the Karolinska University Hospital. Today, 139 patients have so far received this therapy and they constitute a heterogenous group of patients: 102 patients with newly diagnosed GBM, 8 with secondary GBM, and 29 with recurrent GBM. Among these patients, we observed that 102 patients with newly diagnosed GBM who had been treated with valganciclovir had longer median OS (24.1 vs. 13.3 months, *p* < 0.0001) and a higher 2-year survival rate than 231 contemporary controls (49.8% vs. 17.3%). Valganciclovir improved survival in patients with radical or partial resection and with tumors with an unmethylated or methylated O (6)-methylguanine DNA methyltransferase (MGMT) promoter gene, and was safe to use [29]. Patients with secondary GBM receiving valganciclovir survived 19.1 months after their progression to a grade IV tumor, compared to 12.1 months in the controls (*p* = 0.0072) [30].

A promising indication of a possible positive effect of valganciclovir in the treatment of patients with recurrent disease was suggested by Peng et al. in 2016, who showed that combined treatment of valganciclovir and bevacizumab indicated improved survival in 13 patients with recurrent GBM compared to patients treated with bevacizumab alone (*n* = 50); the median OS after recurrence was 13 vs. 8.7 months, respectively (*p* = 0.005). The use of valganciclovir in combination with bevacizumab was reported to be well tolerated [31].

We recently reported that the expression levels of the CMV protein IE were significantly increased in recurrent GBM tissues compared to levels found in matched primary tumor tissue specimens obtained from patients who underwent adjuvant radio and chemotherapy [32]. This observation supports a potential role for anti-CMV therapy in glioblastoma patients with recurrent disease. Here, we present the survival data of the patients with recurrent GBM who were treated with valganciclovir at our institution.

## 2. Materials and Methods

### 2.1. Study Design

This is a retrospective study of the outcome of 29 patients with recurrent GBM who received valganciclovir as add-on therapy to standard treatment between 13 April 2007 and 31 May 2021 at Karolinska University Hospital, in addition to second-line treatment. Twenty-five patients received valganciclovir after their first recurrence and four patients had received the drug after their second recurrence. Age-matched contemporary control patients (*n* = 109) were selected among patients with recurrent GBM with available data who were treated at our institution during the same time period. No active selection was performed regarding survival outcome, therapy, or other clinical characteristics. Most of the patients had asked for the valganciclovir therapy themselves after searching for different therapy options online. The primary end point was survival after recurrence calculated as the interval of time between diagnosis of recurrence and death. The study was approved by the regional ethics committee in Stockholm (Dnr: 2016/1426-31/1). The authors followed all patients throughout the study.

### 2.2. Standard of Care Treatment

Patients’ demographics and clinical characteristics are summarized in Table 1. There is no standard of care for patients with recurrent GBM. Possible alternatives are reoperation, hypofractionated radiation therapy, temozolomide, lomustine (CCNU), bevacizumab, and/or gamma knife treatment. Valganciclovir was given at the standard recommended dose: 900 mg twice daily for 3 weeks followed by 900 mg daily until disease progression or palliative status.

### 2.3. Statistical Analysis

Patients were analyzed for median survival time after recurrence, median overall survival (OS) time after first diagnosis, 1- and 2-year survival rates after recurrence, extent of resection (radical ≥ 90% tumor mass removed, partial or biopsy < 90%), and MGMT methylation status. Other parameters were age, sex, KPS score at the time of recurrence, and IDH1 mutational status. Survival data are presented in graphs as Kaplan–Meier estimates, calculated from the time of surgery or diagnosis of recurrence/progression. For the four patients who started valganciclovir therapy after their second recurrence, survival was calculated from the date of the second recurrence diagnosis to avoid survival bias caused by the interval of time between the first and second recurrence. All statistical hypotheses were two-sided, with a significance level of 5%. Multiple Cox regression analysis was performed to identify possible correlations among clinical and demographic variables in relation to survival. Significance was determined with a log-rank test; *p* < 0.05 was considered significant. Graph Pad Prism (version 9) and SPSS were used for statistical analyses.

## 3. Results

Between 13 April 2007 and 31 May 2021, 29 patients with recurrent GBM received valganciclovir in addition to second- or third-line therapy were included in the analysis. No new or additional toxicity was observed. Contemporary controls were constituted of 109 patients with recurrent disease who were all treated at our institution. Patient characteristics are summarized in Table 1.

### 3.1. Valganciclovir in Patients with Recurrent Disease

**Figure 1:**
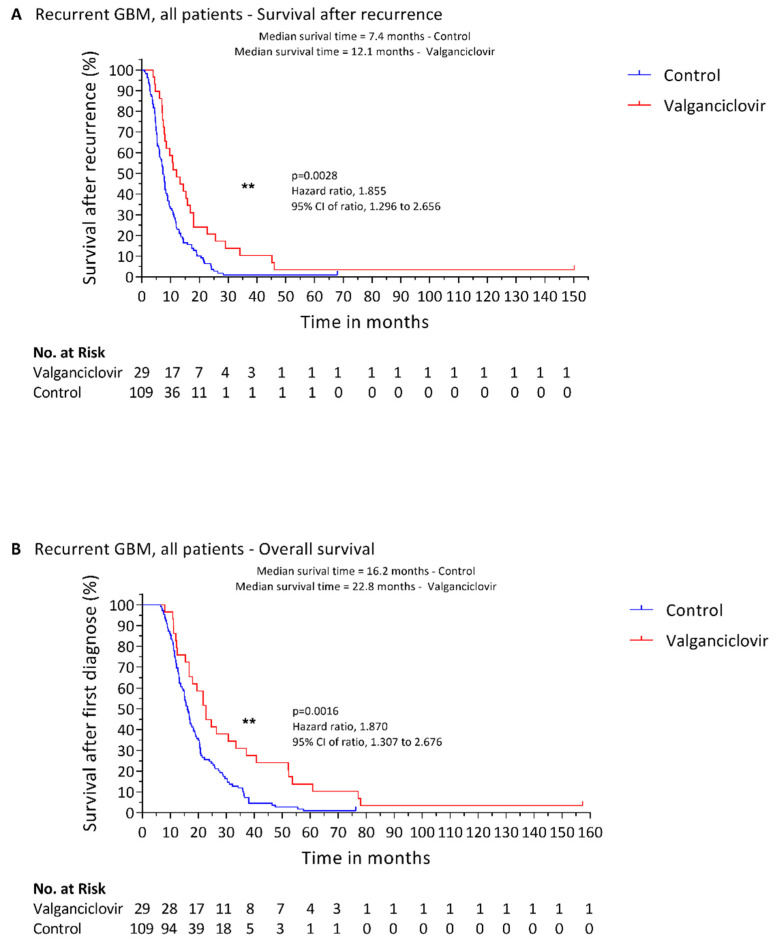
Kaplan–Meier estimates of survival in patients with recurrent glioblastoma treated with valganciclovir and in controls. Estimated survival after recurrence (**A**) and OS after diagnosis (**B**) of all 29 patients with recurrent glioblastoma treated with valganciclovir (red) and of 109 contemporary controls who received similar second-line therapy (blue). Significance is indicated as following: ** (*p* ≤ 0.01).

The median age of patients treated with valganciclovir was 54 years (range, 21 to 72 years) and 65.5% were men. The median age of the 109 controls was 57 years (range 24 to 77 years) and 64% were men. The KPS at the time of recurrence was available for all the patients included in the study and the median KPS value of both treated and control patients was 90. The median time between diagnosis of recurrent disease and start of valganciclovir was 1.8 months (range, 0 days to 7.7 months). The median length of valganciclovir therapy was 8.1 months (range, 1.5 to 147.1 months). Median survival after recurrence was longer in the valganciclovir-treated patients than in the controls (12.1 vs. 7.4 months; *p* = 0.0028) (Figure 1A), as was their OS from primary diagnosis (22.8 vs. 16.2 months; *p* = 0.0016) (Figure 1B). Remarkably, the one-year survival rate was 44.8% in the valganciclovir-treated patients with primary GBM vs. 23.9% among controls (*p* = 0.0122). Two-year survival after recurrence was also much greater in the valganciclovir-treated patients (20.7% vs. 5.5%; *p* = 0.0042). Notably, median time to progression from first diagnosis to recurrence was 7.9 months in controls and 7.4 months in patients subsequently treated with valganciclovir. Thus, the two groups had a comparable clinical prognosis (*p* = 0.1966) before one group had begun valganciclovir therapy, possibly giving them an improved survival chance. IDH1/2 mutational status was available only for 32 patients (24 controls and 6 valganciclovir-treated patients); none of the patients included in this study had a mutation in IDH1/2 genes in accordance to the new definition of glioblastoma of the 2021 WHO Classification of Tumors of the Central Nervous System [33].

### 3.2. Effect of Valganciclovir on Survival according to Second-Line Surgical Intervention

**Figure 2:**
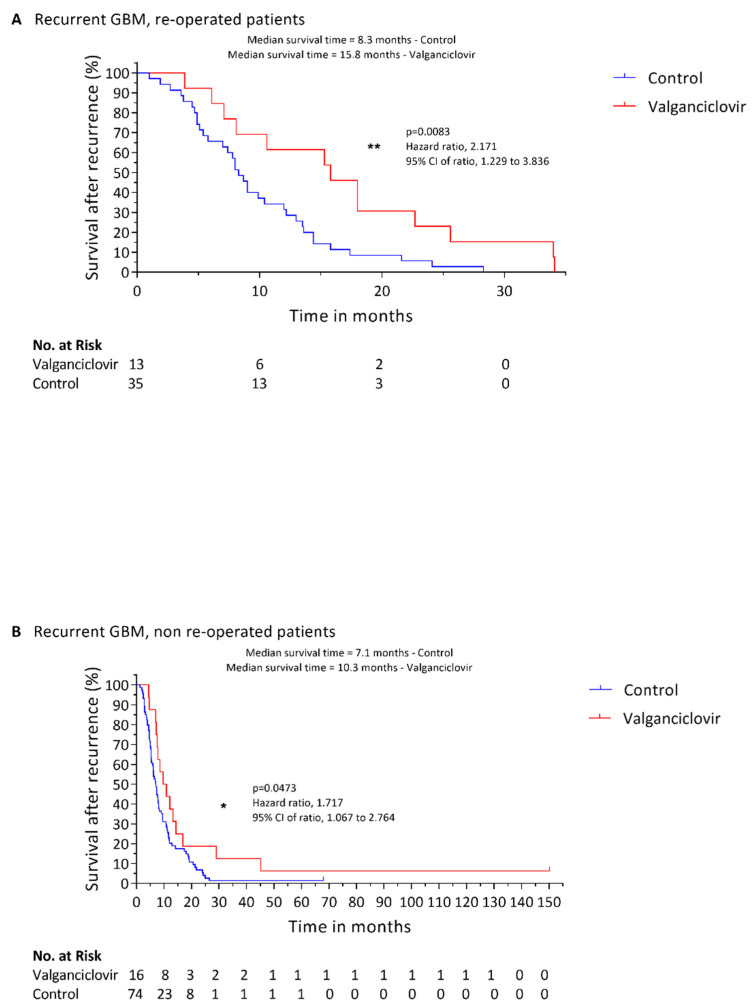
Kaplan–Meier estimates of survival in patients with recurrent glioblastoma treated with valganciclovir according to second-line surgical intervention. Estimated survival after recurrence of 13 valganciclovir-treated patients (red) compared to 35 control patients (blue) who were re-operated at recurrence (**A**). (**B**) includes survival curves of 16 valganciclovir-treated patients (red) compared to 74 control patients (blue) who were not re-operated at recurrence. Significance is indicated as following: * (*p* ≤ 0.05); ** (*p* ≤ 0.01).

In total, 13 patients underwent re-operation before starting valganciclovir therapy. Survival after recurrence was significantly higher in these patients than in 35 re-operated controls (15.8 vs. 8.3 months, respectively; *p* = 0.0083) (Figure 2A). One-year survival rate for re-operated patients was 61.5% for patients treated with valganciclovir and 30% for controls (*p* = 0.0683); 2-year survival rate was 23.1% in patients treated with valganciclovir and 6.7% for controls (*p* = 0.0156). Likewise, among patients who did not undergo a re-operation, 16 who received valganciclovir had significantly longer survival after recurrence than 74 controls (10.3 vs. 7.1 months; *p* = 0.0473) (Figure 2B). One-year survival rate for non-re-operated patients was 43.8% for patients treated with valganciclovir and 20.3% for controls (*p* = 0.0445); 2-year survival rate was 18.8% in patients treated with valganciclovir and 5.4% for controls (*p* = 0.0697). Re-operation provided no significant survival advantage among the controls (*p* = 0.6533) or the valganciclovir-treated patients (*p* = 0.7503).

### 3.3. Effect of Valganciclovir on Survival according to MGMT Methylation Status

**Figure 3:**
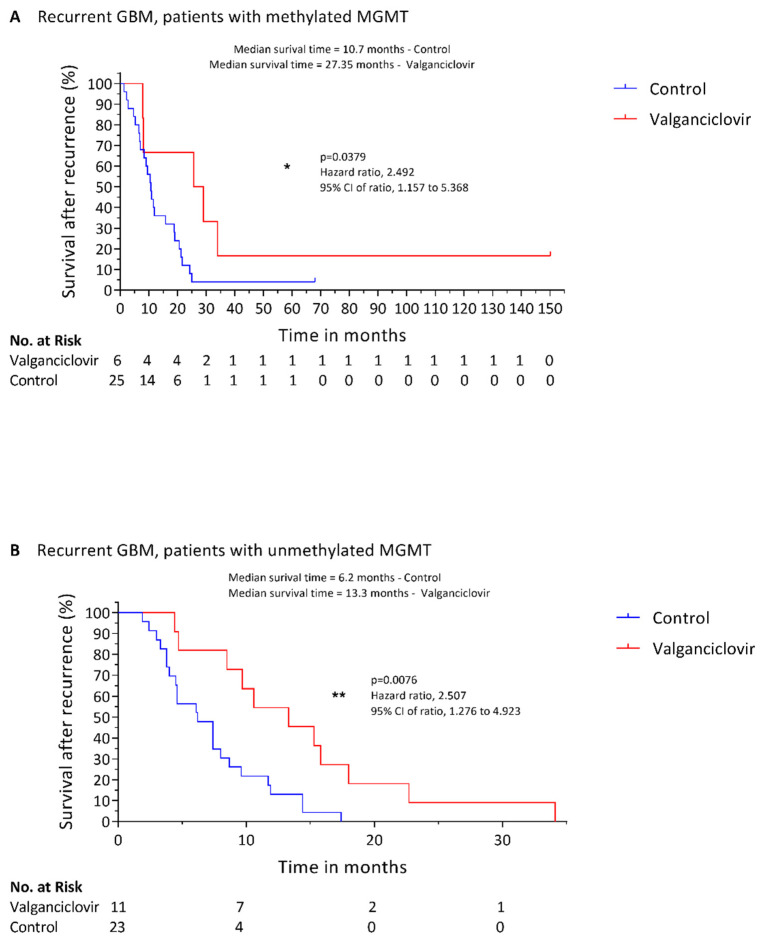
Kaplan–Meier estimates of survival in patients with recurrent glioblastoma treated with valganciclovir according to methylation status of MGMT promoter gene. Estimated survival after recurrence of 6 valganciclovir-treated patients (red) compared to 25 control patients (blue) with methylated MGM promoter gene glioblastoma (**A**) and of 11 valganciclovir-treated patients (red) compared to 23 control patients (blue) with unmethylated MGM promoter gene glioblastoma (**B**). Significance is indicated as following: * (*p* ≤ 0.05); ** (*p* ≤ 0.01).

Data regarding the methylation status of MGMT gene promoter were available for 48 patients in the control group and for 17 patients in the valganciclovir group. In coherence with previously published literature [34], patients in our control cohort having tumors with methylated-MGMT promoter had a longer survival after recurrence compared to patients with unmethylated MGMT promoter status (*p* = 0.003). Among patients with a methylated MGMT promoter, median survival after recurrence was 27.35 months in valganciclovir-treated patients (*n* = 6) compared to 10.7 months in controls (*n* = 25) (*p* = 0.0379) (Figure 3A). One-year survival rate was 66.7% for valganciclovir-treated patients and 36% for controls (*p* = 0.4454), and the 2-year survival rate was 66.7% vs. 12%, respectively (*p* = 0.0369). Valganciclovir appeared to provide a survival advantage also in patients with GBM with an unmethylated MGMT promoter: treated patients (*n* = 11) survived a median of 13.3 months after recurrence compared to 6.2 months of the controls (*n* = 23) (*p* = 0.0076) (Figure 3B). Valganciclovir-treated patients with an unmethylated MGMT promoter had a 1-year survival rate of 54.5% compared to 8.7% for controls (*p* = 0.0052) and the 2-year survival rate was 9.1% vs. 0%, respectively (*p* = 0.0064). In valganciclovir-treated patients the difference in survival between MGMT methylated and unmethylated groups was not statistically significant (*p* = 0.1428), possibly due to the limited number of patients in the two groups.

### 3.4. Multivariable Survival Analysis

Multiple Cox regression analyses implied that methylated MGMT promoter status was the most significant variable positively associated with survival after recurrence (*p* < 0.001) followed by valganciclovir treatment (*p* = 0.004) and KPS score (*p* = 0.012). Among these patients, gender (*p* = 0.921), re-operation (*p* = 0.4), and age (*p* = 0.188) did not correlate with survival (the groups were however age-matched at inclusion). As the number of patients treated with valganciclovir in this study is relatively small (*n* = 29), we decided to address if there could be a possible bias to our positive findings that was related to outliers. We performed a Grubbs’ test on the 29 patients with glioblastoma who were treated with valganciclovir; only one long-term surviving patient was identified as a significant outlier (survival after recurrence was 150.2 months, *p* < 0.05). We then excluded the outlier, and compared the survival after recurrence for patients receiving valganciclovir with the controls. We found that the valganciclovir-treated patients still had significantly longer survival after recurrence than the controls (11.5 months vs. 7.4, *p* = 0.0075).

### 3.5. Effects of Valganciclovir in the Elderly

CMV has been shown to have a variety of negative effects in frail individuals and particularly in the elderly. To address the issue in further depth, we sorted patients included in our study into two groups according to their age, with 60 years of age as a threshold. Of the 29 patients treated with valganciclovir, 11 were older than 60 (median age 61 years) and 18 were younger (median age 51 years). Among the controls, 64 patients were younger than 60 (median age 53) and 45 were older (median age 63). As expected, patients older than 60 tended to have shorter survival compared with younger patients, both in the control group (5.5 vs. 8 months, *p* = 0.138) and in the valganciclovir-treated group (10.9 vs. 13.9 months, *p* = 0.4579), but statistical significance was not reached, possibly due to the small number of patients available for analyses. We then compared survival after recurrence of older patients treated with valganciclovir to older controls and found that patients receiving valganciclovir survived significantly longer (10.9 vs. 5.5 months, *p* = 0.0094). Patients younger than 60 also survived longer after recurrence if they were treated with valganciclovir (13.9 vs. 8.1 months, *p* = 0.0144).

## 4. Discussion

In previous retrospective studies, we have showed that patients with newly diagnosed or secondary GBM survived significantly longer if they had been treated with valganciclovir as an add-on to standard therapy [28,29,30]. In the current study, we carried out a retrospective analysis on survival data for 29 patients with recurrent GBM who received valganciclovir as an add-on to second- or third-line therapy at our institution. Similar to patients with primary GBM [29], we observed a distinctly increased survival rate among patients who had received valganciclovir as an add-on to their second- (*n* = 25) or third-line therapy (*n* = 4), as compared with contemporary control patients. All patients were treated at the same institution by the same clinical team. The median time to progression from first diagnosis to recurrence was 7.9 months in controls and 7.4 months in patients who subsequently were treated with valganciclovir, suggesting that the two groups had a comparable clinical prognosis (*p* = 0.1966) before one group had begun valganciclovir therapy, which appeared to give them an improved survival chance (12.1 vs. 7 months after recurrence, *p* = 0.0028). Thus, although we observed no difference in the time to tumor recurrence between patients who were later treated with valganciclovir or not, patients who received valganciclovir after their recurrence showed significantly enhanced survival rates, as compared to control patients.

The positive effect of valganciclovir therapy seemed to be most prominent in those who underwent surgery to lower their tumor burden after diagnosis of recurrent disease. Patients who were re-operated tended to have a longer median survival after recurrence; 15.8 months compared with 10.3 months in those who did not undergo surgery, but the difference was not significant (*p* = 0.7503), possibly due to low patient numbers. There was also no significant difference in survival between patients who underwent surgery or not among those who did not receive valganciclovir treatment. The median survival after recurrence was 8.3 months in patients undergoing surgery, and 7.1 months in those who did not undergo surgery (*p* = 0.6533). Treatment with valganciclovir doubled the chance of being alive at one year and tripled the chance of being alive at 2 years after recurrence both in re-operated and in non-re-operated patients.

Several other factors such as age, molecular phenotype, and other second-line treatment could affect the treatment results. To avoid possible bias in regard to age, the controls were age-matched with the valganciclovir-treated patients at inclusion before survival analyses (median age 57 vs. 54 years). We found that methylated MGMT promoter status was the most significant variable positively associated with survival after recurrence (*p* < 0.001) followed by valganciclovir treatment (*p* = 0.004). Gender or re-operation did not correlate with survival. Regarding other second-line treatments, the large majority of patients in both groups received CCNU (82.6% of controls and 82.8% of valganciclovir-treated patients) and Gamma-knife treatment in similar proportions (11.9% of controls and 13.8% of valganciclovir-treated patients). More patients in the valganciclovir-treated group received Bevacizumab (44.8% vs. 28.4% in controls, respectively); however, this drug, although effective in reducing tumor-associated edema and deriving symptoms, has not been able to improve survival in randomized studies [13].

A larger proportion of patients in the control group had the favorable-for-survival methylated MGMT promoter (52.1% vs. 35.3%). It is well known that patients with the molecular phenotype unmethylated MGMT promoter status [35] respond poorly to therapies as they have an active MGMT enzyme that will prevent the anticipated effects of temozolomide therapy, for example. The MGMT gene encodes for the enzyme O (6)-methylguanine DNA methyltransferase; when the promoter is methylated the gene is silenced. In its unmethylated state, DNA damage induced by cytotoxic drugs can rapidly be repaired by MGMT and thereby the desired cytotoxic effects induced by alkylating drugs such as temozolomide and CCNU are inhibited [36]. Patients with recurrent GBM who underwent valganciclovir treatment appeared to have prolonged survival regardless of their methylation status in the MGMT promoter. Valganciclovir therapy thereby shows indications of a positive effect on survival also in patients with unmethylated MGMT promoter status, who are arguably more resistant to treatments currently in use, such as alkylating agents. This is in coherence with the results of our previous study, where we showed that both patients with a methylated and unmethylated MGMT promoter status benefitted from valganciclovir therapy [29].

The KPS score is an important score relevant to the survival of patients undergoing oncological therapies [37]. In this study, the patients’ KPS scores were, as expected, associated with survival (*p* = 0.012). Importantly, patients treated with valganciclovir and patients in the control group had the same median KPS at the time of recurrence, thus significantly reducing the risk of having selected patients with higher possibility of surviving longer before their start of anti-viral therapy.

Goerig and colleagues recently demonstrated that patients with brain tumors who were CMV IgG positive and underwent chemo and radiotherapy often had reactivated latent CMV [38,39]. It was proposed that the reactivated infection was primarily caused by radiation, as also patients with brain metastases who were not treated with chemotherapy reactivated CMV. If left untreated, the patients’ prognoses were very poor. These findings are coherent with the data reported by Foster et al., showing a longer OS in patients with GBM who were CMV seronegative and should not be at risk for CMV reactivation [40]. It is therefore possible that, for patients with GBM, undergoing radiotherapy reactivates a clinically relevant CMV infection and that valganciclovir therapy may be effective in treating encephalitis symptoms and clinical disease, which may affect the prognosis. These observations are in line with our previously published data demonstrating that CMV is present in brain metastases of colon and breast cancer, and that higher CMV activity is associated with worse prognosis [41,42]. Furthermore, we recently found that radiation mimicking drugs induced expression of a set of transcription factors that can activate the CMV immediate early promoter [32]. This study provides experimental evidence that may explain why DNA damage, induced by radio-chemotherapy, can induce reactivation of a clinically relevant CMV infection. In support of this hypothesis, we found that recurrent GBM tissue specimens obtained post radio-chemotherapy had higher levels of CMV IE protein expression, as compared with primary tumors from the same patients. Thus, radiation induced damage may reactivate CMV in patients, and valganciclovir may prevent this and positively influence survival chances among these patients. As patients with brain tumors and CMV viremia who exhibited encephalitis-like symptoms had a poor prognosis, diagnosing CMV reactivation following radiation therapy in GBM patients is therefore important to ensure affected patients can be treated appropriately.

Once reactivated, CMV can affect the aggressiveness of tumor cells and promote development of recurrent disease by affecting all the hallmarks of cancer. A persistent CMV infection may be more frequent in older patients and, due to the virus effects on their immune system, this virus may place a higher burden on older patients. Under such circumstances, the effect of valganciclovir may be enhanced in older patients. To address this issue, we examined the effect of valganciclovir in patients younger and older than 60. We observed an increase in survival after recurrence in both patients younger than 60 and older patients who received valganciclovir treatment, but this treatment seemed to be even more significant in patients older than 60 when comparing *p* values to younger patients (*p* = 0.0094 vs. *p* = 0.0144). This observation suggests a possible enhanced effect of valganciclovir in elderly patients. However, the data should be taken with caution due to the limited number of patients and the lack of data concerning CMV status. These factors will be studied in the ongoing randomized clinical trial, VIGAS2, evaluating the effect of valganciclovir in patients with primary glioblastoma.

Except for this potentially promising treatment option, the situation for patients with GBM is very concerning. A number of therapies have been evaluated in the attempt to find new therapy options for patients with GBM. These include innovations for radiotherapy, strategies to overcome blood–tumor barrier for delivery of chemotherapy, and innovations for the delivery of anti-cancer drugs [43]. Techniques and drugs have aimed to locally destroy the tumor, and different immunotherapy and cell-based therapy protocols have been evaluated along with gene therapy-based attempts [43]. The intratumoral heterogeneity of GBMs is high, which is likely a reason for why personalized/precision medicine has so far provided limited success for these patients. To date, the many attempts to develop new therapy protocols for patients with GBM have not lead to any major breakthroughs and the prognosis for patients with GBM remains utterly poor. Tumor-treating fields have shown improved survival in patients with newly diagnosed GBM [44], but conflicting data were reported for patients with recurrent disease [45,46]. Thus, the situation for these patients is even worse and has not led to a standardized second-line therapy protocol.

## 5. Conclusions

Here, we demonstrate promising prolonged survival times in patients with recurrent GBM who were treated with valganciclovir as an add-on to second/third-line therapy. This treatment is well tolerated, with few side effects after many years on this treatment [29]. So far, we have treated 139 patients with GBM with valganciclovir and included 85 patients in a randomized placebo-controlled study (NCT04116411). No new side effects have been observed, and the longest treated patient has today received the drug for over 13 years. Although the number of valganciclovir-treated patients with recurrent GBM is only 29 in the current study and the results should be considered with caution, the effect is similar as we reported for patients with newly diagnosed and secondary GBM, and was statistically significant, even after removal of one identified outlier. This lowers the risk of a random effect by chance. An intrinsic limitation of this study is represented by its retrospective nature, with the contemporary controls receiving baseline therapy rather than a placebo drug. We limited confounding factors consequent from the lack of double blinding by performing age matching and subgroup analyses for relevant clinical characteristics. Other study limitations include potential selection bias as patients (or their relatives) selected themselves to opt for this therapy and they may hence be in better clinical condition as they were able to seek new therapy options themselves. Nevertheless, their KPS scores were similar to those of controls, and they had similar times to recurrence as control patients before they started valganciclovir therapy. A strength of the study is that the patients were treated by the same physicians who followed them throughout the study and most patients in both groups received similar baseline therapy.

As valganciclovir may have a potential positive effect on patients with recurrent GBM who lack any other effective treatment option, it is important to evaluate this well-tolerated and potentially effective therapy in randomized clinical trials. If valganciclovir treatment turns out to be effective, it should be promptly implemented in current treatment protocols.

## Figures and Tables

**Table 1:** Demographic and clinical characteristics.

	Recurrent Disease	
Characteristics	Controls ^1^ (*n* = 109)	Valganciclovir (*n* = 29)
Age, years		
Median	57	54
Range	24–77	21–72
Sex		
Female	39 (35.8%)	10 (34.5%)
Male	70 (64.2%)	19 (65.5%)
Race		
Caucasian	109 (100%)	29 (100%)
MGMT promoter status		
Methylated	25 (52.1%)	6 (35.3%)
Unmethylated	23 (47.9%)	11 (64.7%)
IDH1 mutational status		
Mutated	0 (0%)	0 (0%)
Wild-type	24 (100%)	6 (100%)
KPS score		
Median	90	90
Range	70–100	70–100
Tumor location		
Temporal	44 (40.4%)	13 (44.8%)
Frontal	28 (25.7%)	7 (24.1%)
Parietal	25 (22.9%)	4 (13.8%)
Occipital	6 (5.5%)	3 (10.4%)
Other	6 (5.5%)	2 (6.9%)
Primary treatment		
Surgery		
Radical resection	89 (81.7%)	20 (69%)
Partial resection or biopsy	20 (18.3%)	9 (31%)
Concomitant radio-chemotherapy	98 (89.9%)	26 (89.7%)
Second-line therapy		
Re-operated	35 (32.1%)	13 (44.8%)
Non-re-operated	74 (67.9%)	16 (55.2%)
Gamma-knife treatment	13 (11.9%)	4 (13.8%)
CCNU	90 (82.6%)	24 (82.8%)
Bevacizumab	31 (28.4%)	13 (44.8%)

^1^ Controls received standard of care treatment.

## Data Availability

The data that support the findings of this study are available on request from the corresponding author. The data are not publicly available due to privacy or ethical restrictions.
